# Supplementary material for: A scoping review on the association between early childhood caries and life on land: The Sustainable Development Goal 15
Source: PLoS One. 2024 Jul 11;19(7):e0304523. doi: 10.1371/journal.pone.0304523 (PMC11239008; doi:10.1371/journal.pone.0304523)
Supplement: S1 File — (DOCX) [file pone.0304523.s002.docx]

| History Count | Search Terms | Results |
| --- | --- | --- |
| 3 | TITLE-ABS-KEY ( ( terrestrial  OR  land  OR  inland  OR  freshwater )  AND  ( biodivers*  OR  {species richness} OR bioeconom*  OR  bio-econom*  OR  {biological production}  OR  deforest*  OR  desertif*  OR  {earth system} OR {ecological resilience}  OR  ecosystem*  OR  eco-system*  OR  {trophic cascade}  OR  {trophic level}  OR  {trophic web}  OR  {threatened species}  OR  {endangered species}  OR  {extinction risk}  OR  {extinction risks} OR poach* OR {wildlife product}  OR  {wildlife products}  OR  {wildlife traffic}  OR  {wildlife market} OR {wildlife markets}  OR  {wildlife trafficking}  OR  {invasive species}  OR  {alien species}  OR  {land uses}  OR  {land use}  OR  {land uses}  OR  {land degradation}  OR  {soil degradation} OR {LULUCF} OR *forest* OR  {land conservation} OR wetland* OR mountain* OR  dryland*  OR  {mountainous cover}  OR  {protected area} OR {protected areas} OR  {REDD}  OR  {forest management}  OR  {silviculture}  OR  {timber harvest}  OR  {illegal logging}  OR  {slash-and-burn}  OR  {fire-fallow cultivation} OR  {tree cover}  OR  {soil restoration}  OR  {land restoration} OR  {drought}  OR  {sustainable land management}  OR  {mountain vegetation}  OR  {habitat restoration} OR {Red List species}  OR  {Red List Index} OR {extinction wave}  OR  {habitat fragmentation} OR {habitat loss} OR {Nagoya Protocol on Access to Genetic Resources} OR {genetic resources}  OR  {biological invasion} OR {biodiversity-inclusive}  OR  {forest stewardship council} OR {rainforest alliance}  OR  {forest certification}  OR  {forest auditing} OR {ecotourism}  OR  {community-based conservation}  OR  {community based conservation}  OR  {human-wildlife conflict} ) ) AND  KEY ( dental  AND caries ) | 8 |
| 2 | KEY ( dental  AND caries ) | 64,392 |
| 1 | TITLE-ABS-KEY ( ( terrestrial  OR  land  OR  inland  OR  freshwater )  AND  ( biodivers*  OR  {species richness} OR bioeconom*  OR  bio-econom*  OR  {biological production}  OR  deforest*  OR  desertif*  OR  {earth system} OR {ecological resilience}  OR  ecosystem*  OR  eco-system*  OR  {trophic cascade}  OR  {trophic level}  OR  {trophic web}  OR  {threatened species}  OR  {endangered species}  OR  {extinction risk}  OR  {extinction risks} OR poach* OR {wildlife product}  OR  {wildlife products}  OR  {wildlife traffic}  OR  {wildlife market}  OR  {wildlife markets} OR  {wildlife trafficking}  OR  {invasive species}  OR  {alien species}  OR  {land uses}  OR  {land use}  OR  {land uses}  OR  {land degradation} OR  {soil degradation}  OR  {LULUCF}  OR  *forest*  OR  {land conservation} OR wetland* OR mountain* OR dryland*  OR  {mountainous cover}  OR  {protected area}  OR  {protected areas}  OR  {REDD}  OR  {forest management}  OR  {silviculture}  OR  {timber harvest}  OR  {illegal logging}  OR  {slash-and-burn}  OR  {fire-fallow cultivation}  OR  {tree cover}  OR  {soil restoration}  OR  {land restoration}  OR  {drought}  OR  {sustainable land management}  OR  {mountain vegetation}  OR  {habitat restoration}  OR  {Red List species}  OR  {Red List Index} OR {extinction wave}  OR  {habitat fragmentation}  OR  {habitat loss}  OR  {Nagoya Protocol on Access to Genetic Resources} OR  {genetic resources}  OR  {biological invasion}  OR  {biodiversity-inclusive}  OR  {forest stewardship council}  OR  {rainforest alliance}  OR  {forest certification}  OR  {forest auditing}  OR  {ecotourism}  OR  {community-based conservation}  OR  {community based conservation}  OR  {human-wildlife conflict} ) ) | 431,808 |

Search strategy for Pubmed

| # | Query | Search Details | Results |
| --- | --- | --- | --- |
| 7 | #1 AND #5 | ("Ponds"[MeSH Terms] OR "Lakes"[MeSH Terms] OR "Water"[MeSH Terms] OR "Fresh Water"[MeSH Terms]) AND ("Biodiversity"[MeSH Terms] OR "species richness"[All Fields] OR "bioeconom*"[All Fields] OR "bio econom*"[All Fields] OR "Conservation of Natural Resources"[MeSH Terms] OR "ecological resilience"[All Fields] OR "Grassland"[MeSH Terms] OR "Tundra"[MeSH Terms] OR "Eutrophication"[MeSH Terms] OR "Taiga"[MeSH Terms] OR "Ecosystem"[MeSH Terms] OR "Forests"[MeSH Terms] OR "Introduced Species"[MeSH Terms] OR "Wildlife Trade"[MeSH Terms] OR "Endangered Species"[MeSH Terms] OR "Natural Resources"[MeSH Terms] OR "Environmental Restoration and Remediation"[MeSH Terms] OR "Soil"[MeSH Terms] OR "LULUCF"[All Fields] OR "Forests"[MeSH Terms] OR "Forestry"[MeSH Terms] OR "Rainforest"[MeSH Terms] OR "Trees"[MeSH Terms] OR "Wetlands"[MeSH Terms] OR "mountain*"[All Fields] OR "dryland*"[All Fields] OR "protected area"[All Fields] OR "UN-REDD"[All Fields] OR ("silvicultural"[All Fields] OR "silviculturally"[All Fields] OR "silviculture"[All Fields]) OR "timber harvest"[All Fields] OR "illegal logging"[All Fields] OR "slash-and-burn"[All Fields] OR "tree cover"[All Fields] OR "Environmental Restoration and Remediation"[MeSH Terms] OR "Droughts"[MeSH Terms] OR "sustainable land management"[All Fields] OR "Red List species"[All Fields] OR "Red List Index"[All Fields] OR "extinction waves"[All Fields] OR "habitat fragmentation"[All Fields] OR "habitat loss"[All Fields] OR "Nagoya Protocol on Access to Genetic Resources"[All Fields] OR "genetic resources"[All Fields] OR "biological invasion"[All Fields] OR "biodiversity-inclusive"[All Fields] OR "forest stewardship council"[All Fields] OR "rainforest alliance"[All Fields] OR "forest certification"[All Fields] OR "ecotourism"[All Fields] OR "community based conservation"[All Fields] OR "human-wildlife conflict"[All Fields]) AND ("Dental Caries"[MeSH Terms] OR ("carie"[All Fields] OR "Dental Caries"[MeSH Terms] OR ("dental"[All Fields] AND "caries"[All Fields]) OR "Dental Caries"[All Fields] OR "caries"[All Fields]) OR ("Dental Caries"[MeSH Terms] OR ("dental"[All Fields] AND "caries"[All Fields]) OR "Dental Caries"[All Fields] OR ("dental"[All Fields] AND "decay"[All Fields]) OR "dental decay"[All Fields])) | 19 |
| 5 | #2 OR #3 OR #4 | "Dental Caries"[MeSH Terms] OR ("carie"[All Fields] OR "Dental Caries"[MeSH Terms] OR ("dental"[All Fields] AND "caries"[All Fields]) OR "Dental Caries"[All Fields] OR "caries"[All Fields]) OR ("Dental Caries"[MeSH Terms] OR ("dental"[All Fields] AND "caries"[All Fields]) OR "Dental Caries"[All Fields] OR ("dental"[All Fields] AND "decay"[All Fields]) OR "dental decay"[All Fields]) | 69,231 |
| 4 | dental decay | "dental caries"[MeSH Terms] OR ("dental"[All Fields] AND "caries"[All Fields]) OR "dental caries"[All Fields] OR ("dental"[All Fields] AND "decay"[All Fields]) OR "dental decay"[All Fields] | 66,642 |
| 3 | Caries | "carie"[All Fields] OR "dental caries"[MeSH Terms] OR ("dental"[All Fields] AND "caries"[All Fields]) OR "dental caries"[All Fields] OR "caries"[All Fields] | 68,051 |
| 2 | "Dental Caries"[Mesh] | "Dental Caries"[MeSH Terms] | 49,926 |
| 1 | ("Ponds"[Mesh] OR "Lakes"[Mesh] OR "Water"[Mesh] OR "Fresh Water"[Mesh]) AND ("Biodiversity"[Mesh] OR "species richness" OR bioeconom* OR bio-econom* OR "Conservation of Natural Resources"[Mesh] OR "ecological resilience" OR "Grassland"[Mesh] OR "Tundra"[Mesh] OR "Eutrophication"[Mesh] OR "Taiga"[Mesh] OR "Ecosystem"[Mesh] OR "Forests"[Mesh] OR "Introduced Species"[Mesh] OR "Wildlife Trade"[Mesh] OR "Endangered Species"[Mesh] OR "Natural Resources"[Mesh] OR "Environmental Restoration and Remediation"[Mesh] OR "Soil"[Mesh] OR LULUCF OR "Forests"[Mesh] OR "Forestry"[Mesh] OR "Rainforest"[Mesh] OR "Trees"[Mesh] OR "Wetlands"[Mesh] OR mountain* OR dryland* OR "protected area" OR "UN-REDD" OR silviculture OR "timber harvest" OR "illegal logging" OR "slash-and-burn" OR "tree cover" OR "Environmental Restoration and Remediation"[Mesh] OR "Droughts"[Mesh] OR "sustainable land management" OR "Red List species" OR "Red List Index" OR "extinction waves" OR "habitat fragmentation" OR "habitat loss" OR "Nagoya Protocol on Access to Genetic Resources" OR "genetic resources" OR "biological invasion" OR "biodiversity-inclusive" OR "forest stewardship council" OR "rainforest alliance" OR "forest certification" OR "ecotourism" OR "community based conservation" OR "human-wildlife conflict") | ("Ponds"[MeSH Terms] OR "Lakes"[MeSH Terms] OR "Water"[MeSH Terms] OR "Fresh Water"[MeSH Terms]) AND ("Biodiversity"[MeSH Terms] OR "species richness"[All Fields] OR "bioeconom*"[All Fields] OR "bio econom*"[All Fields] OR "Conservation of Natural Resources"[MeSH Terms] OR "ecological resilience"[All Fields] OR "Grassland"[MeSH Terms] OR "Tundra"[MeSH Terms] OR "Eutrophication"[MeSH Terms] OR "Taiga"[MeSH Terms] OR "Ecosystem"[MeSH Terms] OR "Forests"[MeSH Terms] OR "Introduced Species"[MeSH Terms] OR "Wildlife Trade"[MeSH Terms] OR "Endangered Species"[MeSH Terms] OR "Natural Resources"[MeSH Terms] OR "Environmental Restoration and Remediation"[MeSH Terms] OR "Soil"[MeSH Terms] OR "LULUCF"[All Fields] OR "Forests"[MeSH Terms] OR "Forestry"[MeSH Terms] OR "Rainforest"[MeSH Terms] OR "Trees"[MeSH Terms] OR "Wetlands"[MeSH Terms] OR "mountain*"[All Fields] OR "dryland*"[All Fields] OR "protected area"[All Fields] OR "UN-REDD"[All Fields] OR ("silvicultural"[All Fields] OR "silviculturally"[All Fields] OR "silviculture"[All Fields]) OR "timber harvest"[All Fields] OR "illegal logging"[All Fields] OR "slash-and-burn"[All Fields] OR "tree cover"[All Fields] OR "Environmental Restoration and Remediation"[MeSH Terms] OR "Droughts"[MeSH Terms] OR "sustainable land management"[All Fields] OR "Red List species"[All Fields] OR "Red List Index"[All Fields] OR "extinction waves"[All Fields] OR "habitat fragmentation"[All Fields] OR "habitat loss"[All Fields] OR "Nagoya Protocol on Access to Genetic Resources"[All Fields] OR "genetic resources"[All Fields] OR "biological invasion"[All Fields] OR "biodiversity-inclusive"[All Fields] OR "forest stewardship council"[All Fields] OR "rainforest alliance"[All Fields] OR "forest certification"[All Fields] OR "ecotourism"[All Fields] OR "community based conservation"[All Fields] OR "human-wildlife conflict"[All Fields]) | 51,520 |

Bottom of Form

Search strategy for Web of Science

| 1 | TS=(Ponds) | 54358 |
| --- | --- | --- |
| 2 | TS=Lakes | 257892 |
| 3 | TS=Water | 3577168 |
| 4 | TS=Fresh Water | 120766 |
| 5 | TS=Biodiversity | 190823 |
| 6 | TS=species richness | 87585 |
| 7 | TS=bioeconom* | 6420 |
| 8 | TS=bio econom* | 14942 |
| 9 | TS=bio-econom* | 1852 |
| 10 | TS=Conservation of Natural Resources | 18668 |
| 11 | TS=ecological resilience | 13338 |
| 12 | TS=Grassland | 77183 |
| 13 | TS=Tundra | 12357 |
| 14 | TS=Eutrophication | 36319 |
| 15 | TS=Taiga | 3777 |
| 16 | TS=Ecosystem | 423984 |
| 17 | TS=Forests | 536012 |
| 18 | TS=Introduced Species | 71251 |
| 19 | TS=Wildlife Trade | 3574 |
| 20 | TS=Endangered Species | 43844 |
| 21 | TS=Natural Resources | 142930 |
| 22 | TS="Environmental Restoration and Remediation" | 7 |
| 23 | TS=Soil | 1006333 |
| 24 | TS=LULUCF | 248 |
| 25 | TS=Forests | 536012 |
| 26 | TS=Forestry | 43600 |
| 27 | TS=Rainforest | 18029 |
| 28 | TS=Trees | 647504 |
| 29 | TS=Wetlands | 79033 |
| 30 | TS=mountain | 200495 |
| 31 | TS=dryland | 10258 |
| 32 | TS=protected area | 99199 |
| 33 | TS=UN-REDD | 61 |
| 34 | TS=silvicultural | 6740 |
| 35 | TS=silviculturally | 103 |
| 36 | TS=silviculture | 4312 |
| 37 | TS=timber harvest | 7510 |
| 38 | TS=illegal logging | 1240 |
| 39 | TS=slash-and-burn | 1479 |
| 40 | TS=tree cover | 39684 |
| 41 | TS=Droughts | 140231 |
| 42 | TS=sustainable land management | 21639 |
| 43 | TS=Red List species | 8294 |
| 44 | TS=Red List Index | 720 |
| 45 | TS=extinction waves | 6575 |
| 46 | TS=habitat fragmentation | 22582 |
| 47 | TS=habitat loss | 31498 |
| 48 | TS=Nagoya Protocol on Access to Genetic Resources | 165 |
| 49 | TS=genetic resources | 61913 |
| 50 | TS=biological invasion | 37955 |
| 51 | TS=biodiversity-inclusive | 5 |
| 52 | TS=forest stewardship council | 464 |
| 53 | TS=rainforest alliance | 100 |
| 54 | TS=forest certification | 2069 |
| 55 | TS=ecotourism | 6036 |
| 56 | TS=community based conservation | 18356 |
| 57 | TS=human-wildlife conflict | 2444 |
| 58 | #4 OR #3 OR #2 OR #1 | 3740824 |
| 59 | #5 OR #57 OR #56 OR #55 OR #54 OR #53 OR #52 OR #51 OR #50 OR #49 OR #48 OR #47 OR #46 OR #45 OR #44 OR #43 OR #42 OR #41 OR #40 OR #39 OR #38 OR #37 OR #36 OR #35 OR #34 OR #33 OR #32 OR #31 OR #30 OR #29 OR #28 OR #27 OR #26 OR #25 OR #24 OR #23 OR #22 OR #21 OR #20 OR #19 OR #18 OR #17 OR #16 OR #15 OR #14 OR #13 OR #12 OR #11 OR #10 OR #9 OR #8 OR #7 OR #6 | 2935335 |
| 60 | #59 AND #58 | 684391 |
| 61 | TS=Dental Caries | 34510 |
| 62 | TS=Dental decay | 6116 |
| 63 | TS=caries | 48723 |
| 64 | #63 OR #62 OR #61 | 50216 |
| 65 | #64 AND #60 | 87 |

- WOS.SCI: 1900 to 2023
- WOS.AHCI: 1975 to 2023
- WOS.BHCI: 2005 to 2023
- WOS.BSCI: 2005 to 2023
- WOS.ESCI: 2005 to 2023
- WOS.ISTP: 1990 to 2023
- WOS.SSCI: 1900 to 2023
- WOS.ISSHP: 1990 to 2023
